# Supplementary material for: An ARF gene mutation creates flint kernel architecture in dent maize
Source: Nat Commun. 2024 Mar 22;15:2565. doi: 10.1038/s41467-024-46955-9 (PMC10960022; doi:10.1038/s41467-024-46955-9)
Supplement: Supplementary file 1 — Supplementary Information files [file 41467_2024_46955_MOESM1_ESM.pdf]

An *ARF* gene mutation creates flint kernel architecture in dent maize

Wang et al.

Supplementary Figures

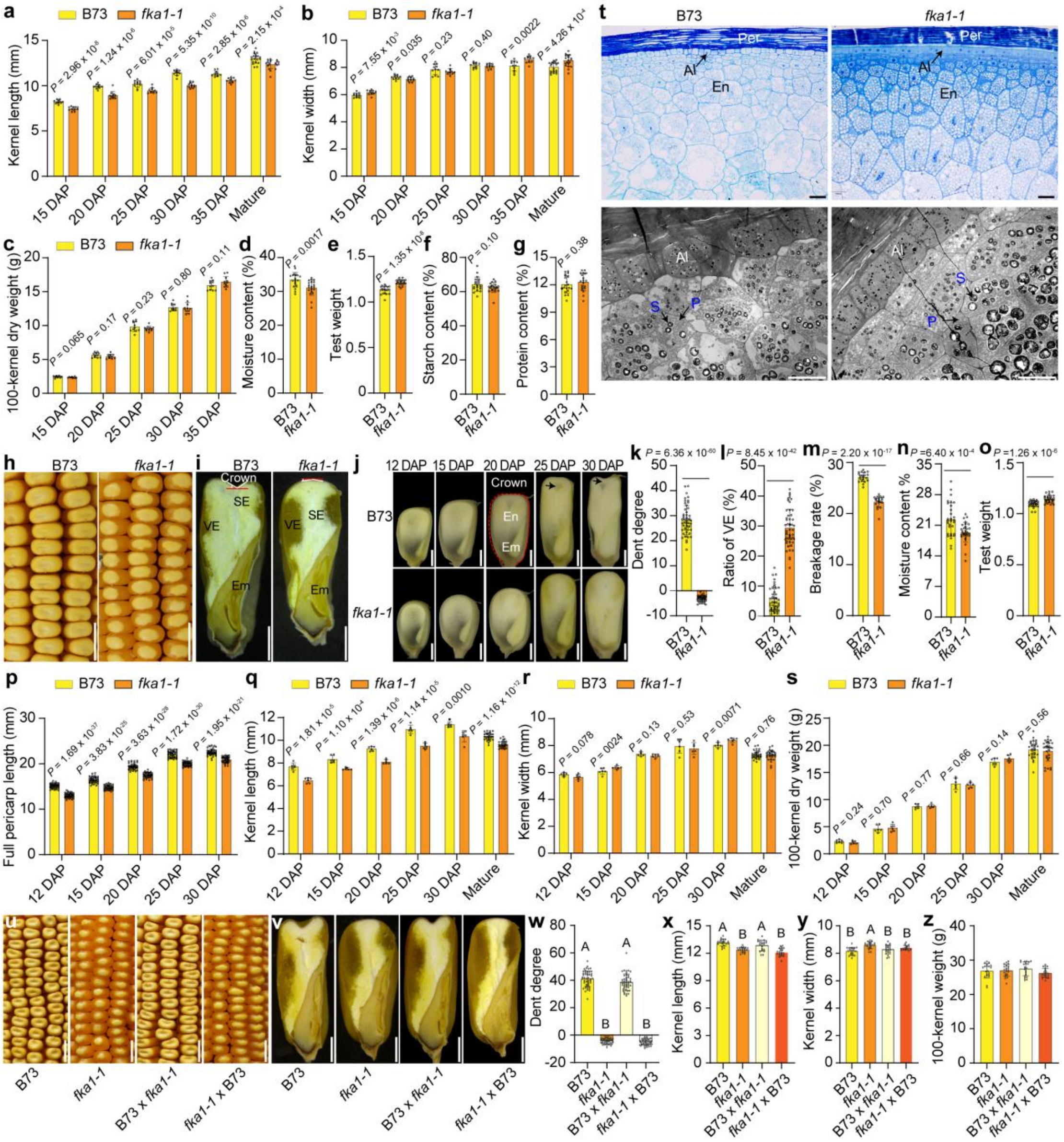

Supplementary Fig.1|Kernel phenotypes of B73, *fka1-1* and their reciprocal crosses. (**a** and **b**) Kernel length (a) and kernel width (b) of B73 and *fka1-1*. Data are mean ± s.d. (*n* = 11 during grain filling, *n* = 20 at maturity). (**c** to **g**) 100-kernel dry weight (c), moisture content (d), test weight (e), total starch content (f) and total protein content (g) in mature kernels. Data are mean ± s.d. (*n* = 20 ears). (**h**) Kernel phenotypes. Scale bar, 1 cm. (**i**) Longitudinal-sections. Scale bar, 2 mm. (**j**) Longitudinal-sections of developing kernels. Black arrows indicate area of incomplete grain filling. Scale bar, 2 mm. (**k**) Kernel crown collapse. Data are mean ± s.d. (*n* = 50 kernels from 20 ears). (**l**) Ratio of vitreous endosperm (VE) area. Data are mean ± s.d. (*n* = 50 kernels from 20 ears). (**m**) Breakage rate. Data are mean ± s.d. (*n* = 20 ears). (**n** and **o**) Kernel moisture content (n) and test weight (o). Data are mean ± s.d. (*n* = 30 ears). (**p**) Pericarp length. Data are mean ± s.d. (*n* = 49 at 12 DAP, *n* = 47 at 15 DAP, *n* = 48 at 20, 25 and 30 DAP). (**q** to **s**) Kernel length (q), kernel width (r) and 100-kernel dry weight (s) during grain filling and at maturity. Data are mean ± s.d.

(*n* = 6 ears during grain filling, 30 ears at maturity). (**t**) Semi-thin sections and TEM observations of the kernel crown regions at 20 DAP. Scale bar in semi-thin sections, 100 μm; scale bar in TEM, 20 μm. (**u-z**) Ears (u), longitudinal-sections (v), degree of kernel crown (w), kernel length (x), kernel width (y) and 100-kernel dry weight (z) of B73, *fka1-1*, B73 x *fka1-1* and *fka1-1* x B73. Scale bar, 1 cm in u; Scale bar, 2 mm in v. In w, data are mean ± s.d. (*n* = 48, *n* = 52, *n* = 45 and *n* = 39, respectively). In x-z, data are mean ± s.d. (*n* = 20 ears). a-g, B73 and *fka1-1* grown in Sanya. h-s, B73 and *fka1-1* grown in Shanghai. Al, aleurone; Em, embryo; En, endosperm; P, protein body; Per, pericarp; S, starch granule; SE, starchy endosperm; VE, vitreous endosperm. Two-tailed Student's *t* tests were used to determine *P* values shown in the a-g, k-s. Letters in w-z indicate significant differences ($P < 0.01$, one-way ANOVA, Tukey's test).

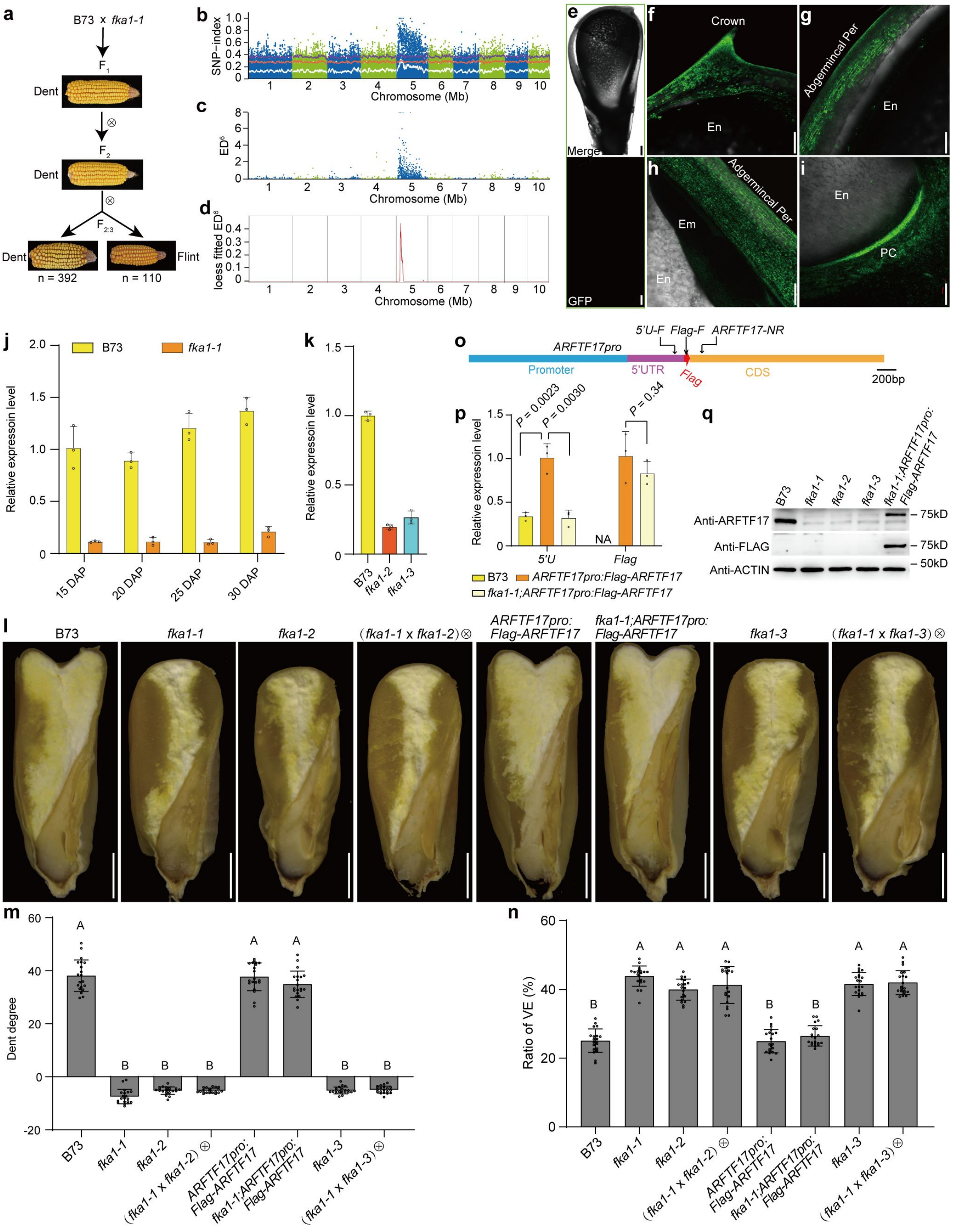

Supplementary Fig.2|Cloning of *fka1-1* by BSA and genetic verification of *fka1-1*. (**a**) A schematic diagram showing the creation of a genetic population for mapping *fka1-1*. (**b**) Mapping by sequencing of *fka1-1*. Ratios of SNP index between dent and flint like pools. (**c**) Distribution of ED^6 across the 10 maize chromosomes. (**d**) Loess fit curve calculated using the data shown in (c). (**e**) *ARFTF17pro:GFP* negative control. Scale bar, 500 µm. (**f** to **i**) Localized magnification of different regions in the *ARFTF17pro:GFP* kernel at 12 DAP pericarp in the kernel crown (f), pericarp on the abgerminal kernel side (g), pericarp on the adgerminal kernel side (h), and the pedicel region (i). Em, embryo; En, endosperm; PC, placenta-chalaza; Per, pericarp. Scale

bar, 100 µm. (**j**) Expression of *ARFTF17* in the pericarp of B73 and *fka1-1* at different periods of development. (**k**) RT-qPCR analysis of *ARFTF17* expression in the pericarp of B73 and that of different *fka1* mutant alleles at 20 DAP. (**l**) Longitudinal-sections of kernels of B73, *fka1-1*, *fka1-2*, *fka1-3*, *ARFTF17pro:Flag-ARFTF17*, *fka1-1; ARFTF17pro:Flag-ARFTF17* and F_2 seeds of *fka1-1* x *fka1-2* and *fka1-1* x *fka1-3.* Scale bar, 2 mm. (**m** and **n**) Kernel crown collapse (m) and ratio of vitreous endosperm (VE) area (n). Data are mean ± s.d. (n = 20). (**o**) Structure of the *ARFTF17pro:Flag-ARFTF17* expression cassette. 5'UTR, the 5' untranslated region of *ARFTF17*; Flag, FLAG tag sequence. Scale bar, 200 bp. (**p**) Detection of *ARFTF17pro:Flag-ARFTF17* and *fka1-1*; *ARFTF17pro:Flag-ARFTF17* by RT-qPCR. *5'U*, the primer pair using the forward *5'U-F* in the 5' UTR and the reverse *ARFTF17-NR* in the first exon of *ARFTF17*; *Flag*, the primer pair using the forward *Flag-F* in the *FLAG* sequence and the reverse *ARFTF17*-NR. (**q**) Immunodetection of ARFTF17 and the FLAG-ARFTF17 fusion protein in the pericarp of B73, *fka1-1, fka1-2*, *fka1-3* and *fka1-1; ARFTF17pro:Flag-ARFTF17* at 15 DAP. ACTIN was used as an internal control. Gene expression levels in j, k, p were normalized to that of *TUA4* (*Zm00001d013367*) and data are mean values ± s.d. (n = 3). Two-tailed Student's t tests were used to determine P values shown in p. Letters in m and n indicate significant differences (P < 0.01, one-way ANOVA, Tukey's test).

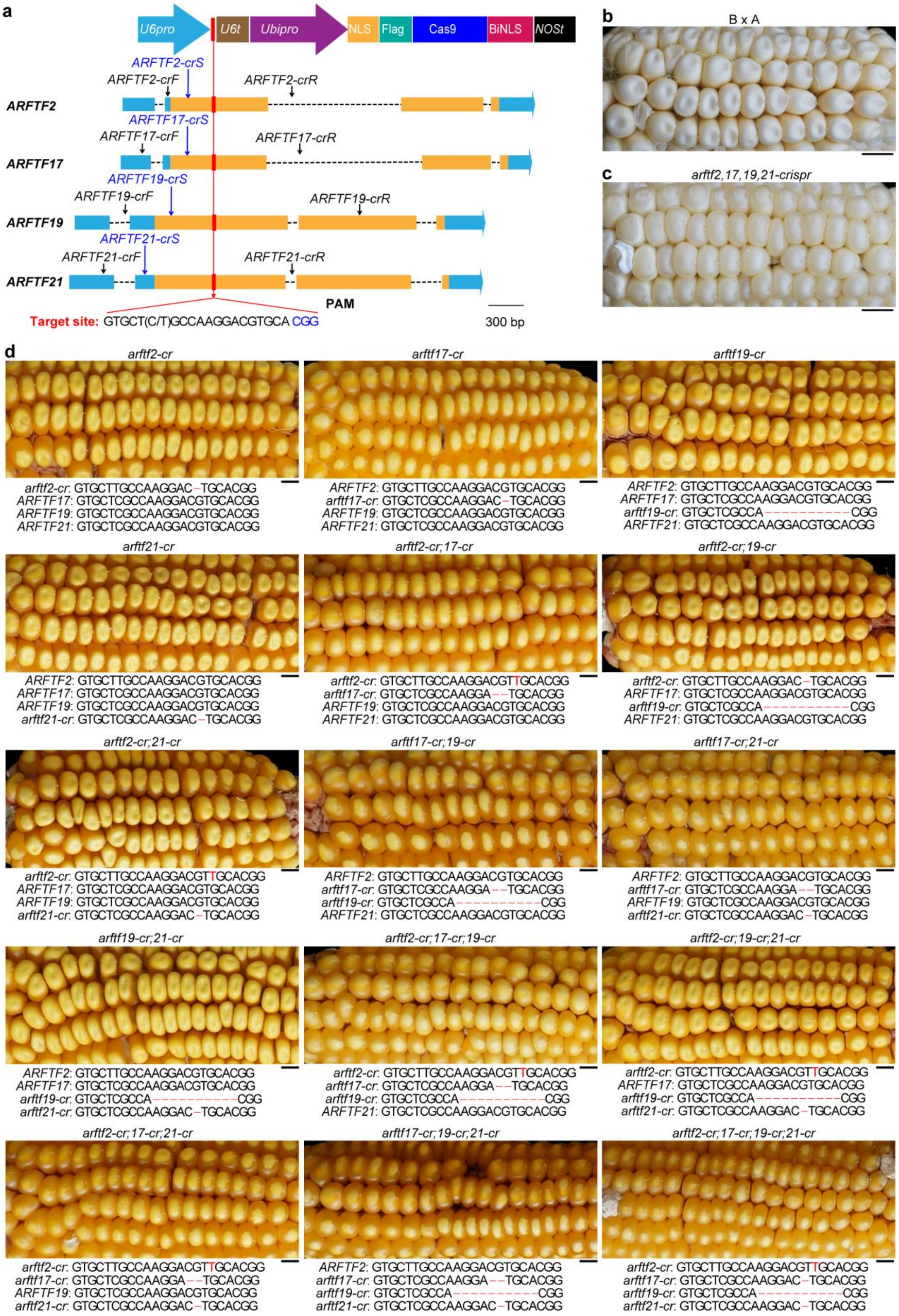

Supplementary Fig.3|Genome editing of the *ARFTF17* and its homologous genes. (**a**) Diagram of the CRISPR/Cas9 construct targeting*ARFTF17* and its homologous genes. The conserved target site is indicated by the red box. Blue boxes indicate 5'UTR and 3'UTR; yellow boxes indicate coding sequences; horizontal dotted

lines indicate introns. The primer pairs (*ARFTF2-crF* + *ARFTF2-crR*, *ARFTF17-crF* + *ARFTF17-crR*, *ARFTF19-crF* + *ARFTF19-crR* and *ARFTF21-crF* + *ARFTF21-crR*) were used to amplify fragments containing edited sites in the four maize *ARFTF* genes. *ARFTF2-crS*, *ARFTF17-crS*, *ARFTF19-crS* and *ARFTF21-crS* were used to sequence the amplified fragments. (**b** and **c**) Ear phenotype of the dent B x A wild type (b) and the edited flint-like *ARFTF2,17,19,21-crispr* mutant (c). Scale bar, 1 cm. (**d**) Ear phenotypes and mutant sites of four single, six double, four triple and one quadruple *ARFTF* mutants. Each mutant site was detected by PCR and sequencing using the primers shown in (a). The mutation in *ARFTF17* and its homologous genes are indicated under the ear. The red lines indicate the missing nucleotides. Scale bar, 1 cm.

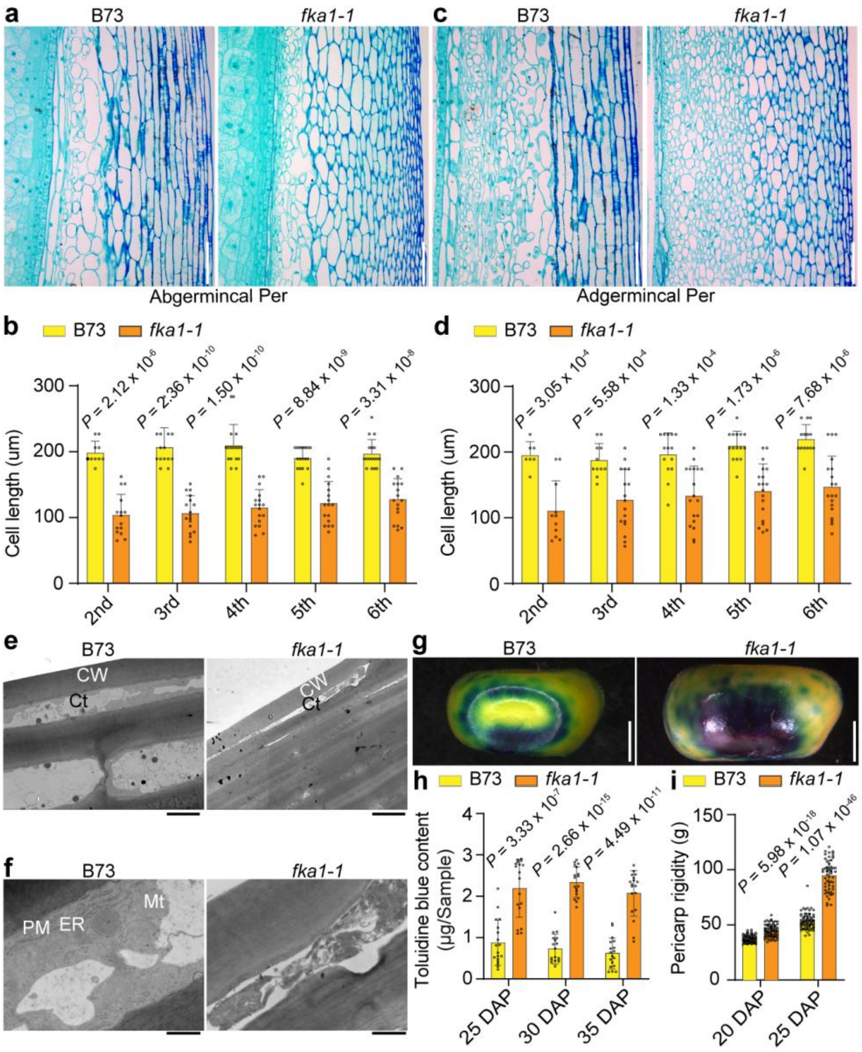

Supplementary Fig.4|Differences in pericarp development of B73 and *fka1-1.* (**a**) Micrographs of semi-thin sections of the pericarp on the abgerminal kernel side of B73 and *fka1-1*. Scale bar, 100 μm. (**b**) Length of pericarp cells on the abgerminal sides in B73 and *fka1-1* kernels. Data are mean ± s.d. (*n* = 11, 14, 17, 17 and 17 in the cell layer of B73, respectively; *n* = 15, 17, 17, 17 and 17 in the cell layer of *fka1-1*, respectively). (**c**) Micrographs of semi-thin sections of the pericarp on the adgerminnal kernel side of B73 and *fka1-1*. Scale bar, 100 μm. (**d**) Length of pericarp cells on the adgerminal sides of B73 and *fka1-1* kernels. Data are mean ± s.d. (*n* = 7, 11, 14, 15 and 14 in the cell layer of B73, respectively; *n* = 11, 17, 18, 19 and 19 in the cell layer of *fka1-1*, respectively). The 2nd, 3rd, 4th, 5th, and 6th represent the second, third, fourth, fifth and sixth cell layer, counting from the outermost cell layer of the pericarp. (**e**) The outermost pericarp cells in B73 and *fka1-1* at 30 DAP. (**f**) Higher resolution of the cells in (e). B73 shows intact organelles and *fka1-1* shows condensed cytoplasm. Scale bar in (e), 5 μm; in (f) 1 μm. Ct, cytoplasm; CW, cell wall; ER, endoplasmic reticulum; Mt, mitochondrion; PM, plasma membrane. (**g**) Kernel crown regions of B73 and *fka1-1* at 35 DAP stained with toluidine blue. Scale bars, 2mm. (**h**) Quantification of toluidine blue stainingin the pericarp of the kernel crown regions of B73 and *fka1-1* at 25, 30and 35 DAP. Data are mean ± s.d. (*n* = 18 kernels).

(**i**) Pericarp rigidity in kernel crown regions of B73 and *fka1-1* at 20 and 25 DAP. Data are mean ± s.d. (*n* = 76, 74 kernels of B73 and *fka1-1* at 20 DAP, and 71, 56 kernels of B73 and *fka1-1* at 25 DAP). Two-tailed Student's *t* tests were used to determine *P* values shown in the b, d, h, i.

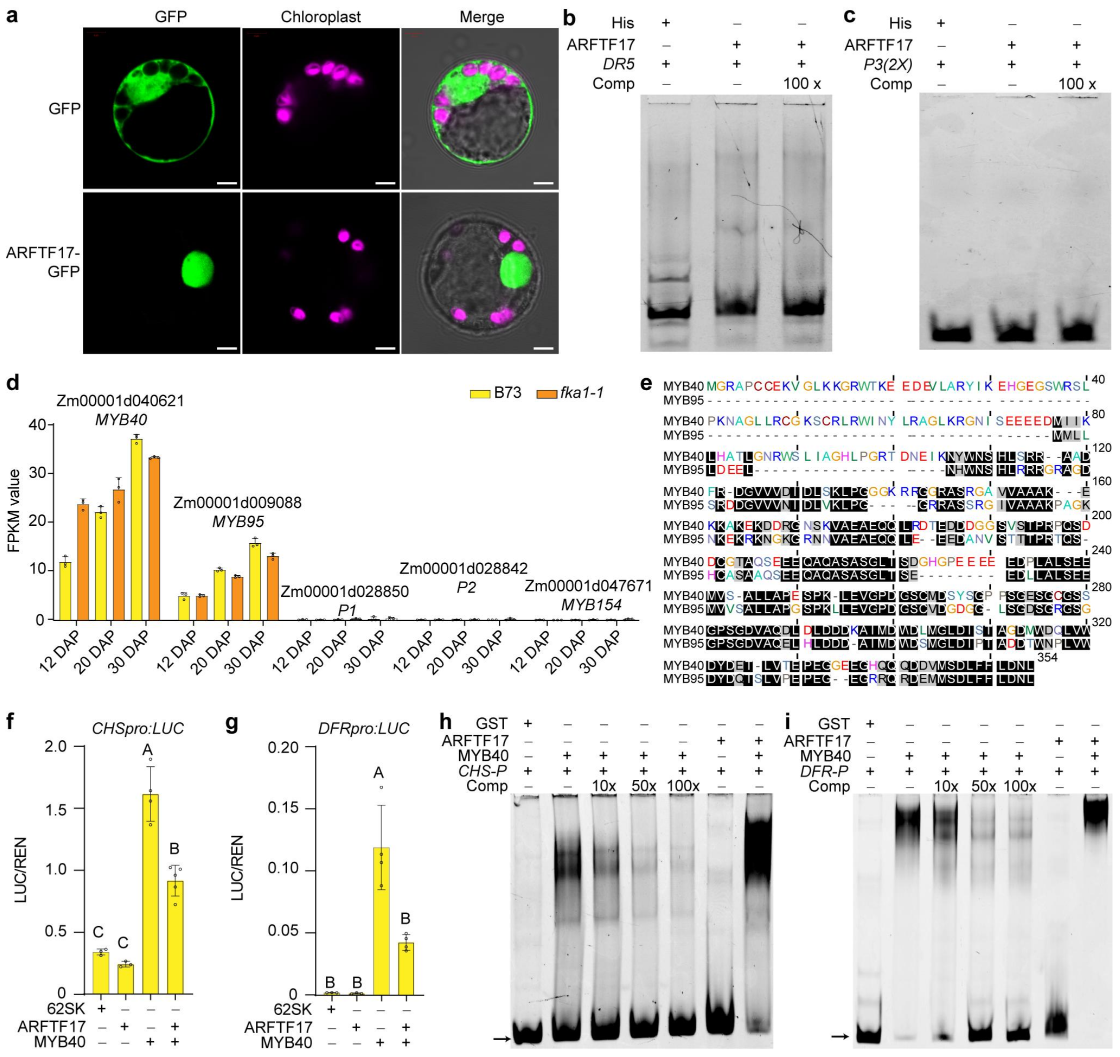

Supplementary Fig.5|MYB40 directly regulates the ARFTF17 downsteam genes. (**a**) Subcellular localization of ARFTF17. To investigate the subcellular localization of ARFTF17, we fused full-length ARFTF17 to the N-terminus of the enhanced green fluorescent protein (eGFP). Transient expression of the fused protein in maize leaf protoplasts revealed its localization in nuclei. Scale bar, 5 µm. (**b** and **c**) EMSA analysis of the DNA binding function of ARFTF17 to the *DR5* promoter (b) and to P3(2x), consisting of two tandem copies of inverted repeats of the TGTCTC element (c). (**d**) FPKM values of *P*-related genes in the whole pericarp tissues of B73 and *fka1-1*. Data are mean ± s.d. ($n = 3$). (**e**) Amino acid sequence alignment of MYB40 and MYB95. Black shaded amino acids represent 100% identical amino acid residues and gray ones indicate the similar amino acid residues. (**f** and **g**) Transactivation in maize leaf protoplasts of *CHS* (f) and *DFR* (g) promoters by MYB40 and repression by ARFTF17. Data are mean ± s.d. ($n = 3$, 3, 4 and 5 in f, respectively; $n = 3$, 3, 4 and 4 in g, respectively). Letters indicate significant differences ($P < 0.01$, one-way ANOVA, Tukey's test). (**h** and **i**) EMSA showing MYB40 binds to the *CHS* (h) and *DFR* (i) promoters. *CHS-P*, the probe containing P1 binding cores positioned between -414 to -291 bp upstream of the start codon in the *CHS* promoter; *DFR-P*, the probe containing P1 binding cores positioned between -255 to -80 bp upstream of the start

codon in the *DFR* promoter.

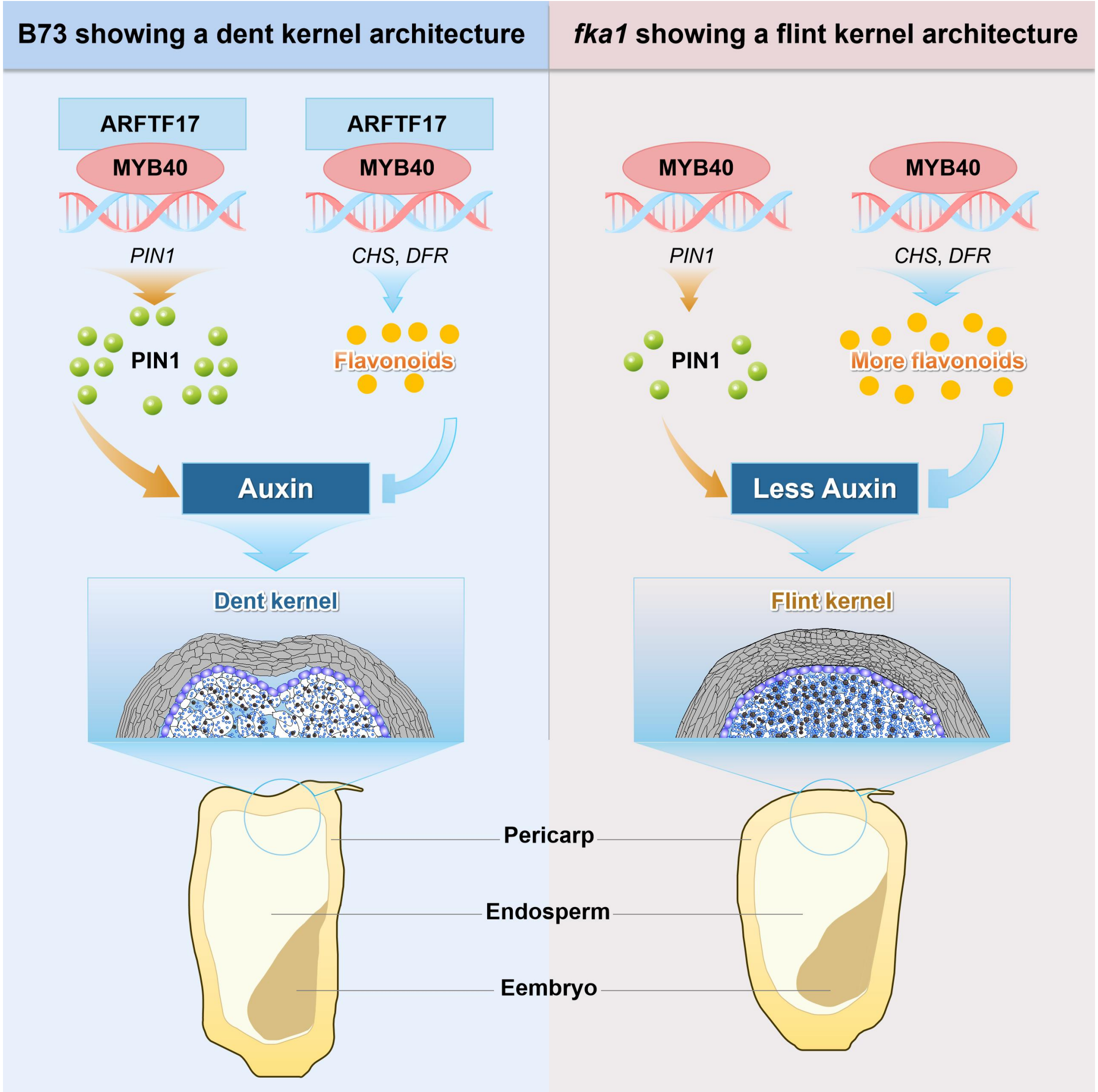

Supplementary Fig.6|Proposed model describing activity of the ARFTF17-MYB40 module regulating pericarp development in maize. *ARFTF17* is highly expressed in the pericarp, where it interacts with MYB40 to exert its repressing function. MYB40 has the dual functions of repressing *PIN1* expression and transactivating genes for flavonoid biosynthesis. Thus, mutation of *ARFTF17* or overexpression of *MYB40* can reduce *PIN1* expression and promote flavonoid biosynthesis. Together, these effects lead to decreased auxin accumulation and a shorter pericarp, thereby creating a flint kernel architecture.

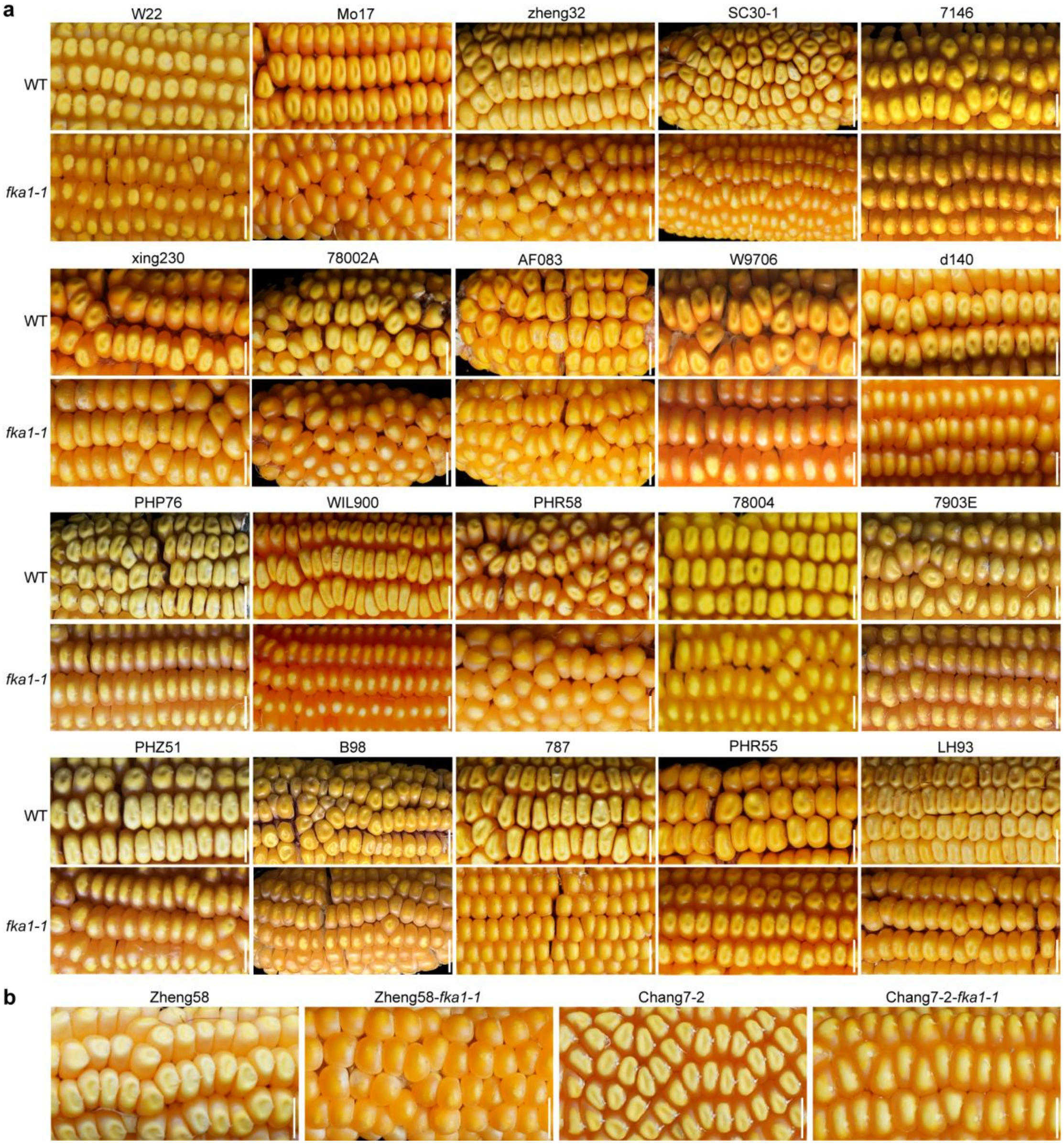

Supplementary Fig.7|Flint-like kernel phenotypes of *fka1-1* in different dent genetic backgrounds. (**a**) Phenotypes of 20 dent inbred lines expressing *fka1-1. fka1-1* was crossed with 20 dent inbred lines and the ears of F_2 plants with homozygous *fka1-1* were compared with the wild-type ears. Scale bar, 1 cm. (**b**) Phenotypes of Zheng58-*fka1-1* and Chang7-2-*fka1-1*. *fka1-1* was introgressed into Zheng58 and Chang7-2 by backcrossing for four generations. Homozygous Zheng58-*fka1-1* and Chang7-2-*fka1-1* were obtained by self-pollination. Scale bar, 1 cm.

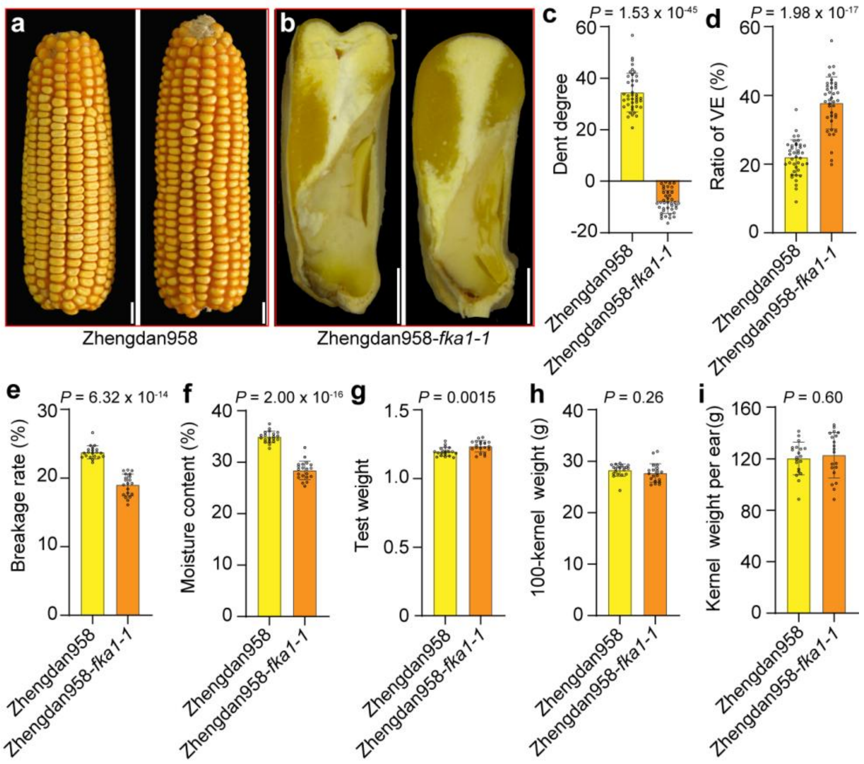

Supplementary Fig.8|Effect of *fka1-1* on the Zhengdan958 hybrid grown in Shanghai. (**a**) Ear phenotypes of Zhengdan958 and Zhengdan958-*fka1-1* hybrids. Scale bars, 1cm. (**b**) Longitudinal-sections of Zhengdan958 and Zhengdan958-*fka1-1* kernels*.* Scale bars, 2 mm. (**c**) Measurement of kernel crown dent degree in Zhengdan958 and Zhengdan958-*fka1-1*. Data are mean ± s.d. (*n* = 40 kernels from 20 ears). Scale bar, 1 cm. (**d**) Ratio of vitreous endosperm (VE) area to the total endosperm. Data are mean ± s.d. (*n* = 40 kernels from 20 ears). (**e** to **i**) Breakage rate of kernels (e), kernel moisture content (f), test weight (g), one hundred-kernel dry weight (h) and kernel weight per ear (i) of Zhengdan958 and Zhengdan958-*fka1-1*. Data are mean ± s.d. (*n* = 20 ears). Two-tailed Student's *t* tests were used to determine *P* values shown in the c-i.

Supplementary Table 1. Synthetic sequences and Primers used in this study.

Name	Sequence (5'-3')	Usage
ARFTF-CRISPR	TTATAACCGAGCCGCAAGCACCGAATTGTGCTC<br>GCCAAGGACGTGCAGTTTTAGAGCTAGAAATAG<br>CAAGTTAAAATAAGGCTAGTCCGTTATCAACTTG<br>AAAAAGTGGCACCGAGTCGGTGCTTTTTTTTCTA<br>GA	CRISPR vector construction
MYB40-RNAi	ggatccCCTGATCGCCGGTCACTTGCCCGGTCGAA<br>CAGACAACGAGATCAAAAACTACTGGAACTCGC<br>ACCTGAGCAGGCGGGCGGCCGACTTCCGCGAC<br>GGCGTCGTCGTCGACATCGACCTCAGCAAGCTG<br>CCCGGCGGCGGGAAACGGCGCGGCGGCCGGG<br>CCAGCCGGGGCGCCGTCGTGGCCGCGGCCAAG<br>GAGAAGAAGGcccggggtacgtatataccgcagtactgatacta<br>ctagctagctagcgctacgtgtacatgtgtatgatctggcagcatgcata<br>tatatatatatgtatgcgtccagtccagctgatgatgcctaatttttttttctctt<br>gccacacatgtacgtatgcatgtacgtttctccttgcgtcaattcgcggcg<br>gcgtacgtgtgcagggcgcgccCCTTCTTCTCCTTGGCCG<br>CGGCCACGACGGCGCCCCGGCTGGCCCGGCC<br>GCCGCGCCGTTTCCCGCCGCCGGGCAGCTTGC<br>TGAGGTCGATGTCGACGACGACGCCGTCGCGG<br>AAGTCGGCCGCCCGCCTGCTCAGGTGCGAGTT<br>CCAGTAGTTTTTGATCTCGTTGTCTGTTCGACCG<br>GGCAAGTGACCGGCGATCAGGgagctc	*MYB40-RNAi* vector construction
fka1-1-F	AAGGGTCCTGGTGGGTACAT	
fka1-1-R	TGCTTTAGCGTGGGACTGAC	
fka1-2-F	GTCCTTCAAGAACGCCGACA	
fka1-2-R	CGCTCCCGTGTCTACTACTTCC	
ARFTF2-crF	ACGAAGAGGAGGAGGAGT	
ARFTF2-crR	TCAGGTGGGCTACAAACA	
ARFTF2-crS	CGCTCCCGTGTCTACTA	
ARFTF17-crF	TACATTCCTACTCCGCTTTG	
ARFTF17-crR	CCTGCCCTACCCTCATAC	Mutant identification
ARFTF17-crS	CGCTCCCGTGTCTACTA	
ARFTF19-crF	CCCCTTGCTTTCTTCTCAC	
ARFTF19-crR	GCCGCCGTGGACGCCTTC	
ARFTF19-crS	GTGCCTGGACCCGCAGCTGTGG	
ARFTF21-crF	CCCGAATCCGTCGTTACCC	
ARFTF21-crR	GCTTTGAGATGCCGTCCTT	
ARFTF21-crS	CAATTCCCGCCGAAACCG	
pin1-F	GCGATGCGACTCCAGACAGA	
pin1-R	ACACCTGTTCTGTTCTTGTTGGAC	

FKA1-1-QF	GGCTTGGTGCTATGACTCCGACG	
FKA1-1-QR	CGAACAGCATTATGTGGGGTCTC	
STG1-QF	CGTCCACCCATAGTGCCATGAG	
STG1-QR	ACGGCAAGTAGCACTCAGACAC	
TUA4-QF	GAGGAGGTCGGTGCTGAGTTT	
TUA4-QR	GAACCGACAGAAACATAACACGAT	
Flag-QF	CAAGGATGACGATGACAAGGG	
ARFTF17-QNF	CTGCAGCTGGACAGTGATCG	
ARFTF17-QNR	GCCCTGCGGGAAGTAGTAGA	
MYB40-QF	GGAGGTCACTGCCCAAGAATG	
MYB40-QR	CAGGTGCGAGTTCCAGTAGTTTT	
PIN1-QF	AGTGTTTCTTGGTGCGTCGAT	
PIN1-QR	TGGAGATGAAGTGGAAGGACAG	
Zm00001d017279 PAL-F	AGGAGCTGAACAAGGTGCTC	
Zm00001d017279 PAL-R	TCGAAGTTGGTTACAGGGCG	
Zm00001d017276 PAL-F	ATCAACCAGGGCAAGCACAT	
Zm00001d017276 PAL-R	CACTCACCAGACCTTGCTCA	
Zm00001d017276 PAL-F	TCCTCTACAGCTTCCCAGCA	
Zm00001d017276 PAL-R	GCCCTCGATCTTCACCAAGG	
Zm00001d003016 PAL-F	CAATGAACCTGGGCAAGCAC	
Zm00001d003016 PAL-R	GGGCCCCATCAAGACATCAA	Real-time PCR
Zm00001d003016 PAL-F	ACAAGGTGTTCCTGGCCCTCA	
Zm00001d003016 PAL-R	GCCTGCTTGAGAATACCGAAA	
Zm00001d017274 PAL-F	CGTCAAGTAAAGAACGCCAAGG	
Zm00001d017274 PAL-R	AGAAAGAGCAACGCCACACAC	
Zm00001d017275 PAL-F	AGCTCAACAAGGTGCTCGTGG	
Zm00001d017275 PAL-R	ACTAATAATACAAGGCGACGGTAGA	
Zm00001d016471 C4H-F	GCGCAGCTTCGAGATGGTA	
Zm00001d016471 C4H-R	TATGCAGAAAACGACGCGCA	
Zm00001d052673 CHS-F	TCCAATTCGTCGTCGTCTCG	
Zm00001d052673 CHS-R	ACCACCCCATGCATGCTAAA	
Zm00001d005823 FLS-F	GTCATCCAGGTGAGCCTAGC	
Zm00001d005823 FLS-R	GGACAAACAACCGACGCAAA	
Zm00001d044122 DFR-F	GACCTCGGGTTCACCTTCAG	
Zm00001d044122 DFR-R	TAGCATCATCGACGGGGAGA	
Zm00001d004366 UGT-F	TTTCAAGGCTTCACGAGACAAA	
Zm00001d004366 UGT-R	TGAACGGTAATGACTGACCTAATCT	
Zm00001d052492 UGT-F	CCAGGAAGTGGGCCGACTTT	
Zm00001d052492 UGT-R	GGGTGGGCAATGTTATTAATGTAC	
Zm00001d037383 UGT-F	GACAAGGTGAAGGAGGTTATGGC	
Zm00001d037383 UGT-R	TGTAACAGCAGTCACTTCACTCGTC	

Zm00001d045254 UGT-F	GACGTGTCGGTGCAGAAACTG	
Zm00001d045254 UGT-R	ACAACGTGAGCAGTGCCAGAC	
Zm00001d051529 4CL-F	ATCTCGACAGCATCACTGCC	
Zm00001d051529 4CL-R	GCCCTCGAGAAGCAGTAGTC	
ARFTF17Pro-F	cgacggccagtgccaagcttATCGCCGCTCAGGTGGTCGCTG	Transgenic vector construction
ARFTF17Pro-R	ggggaaattcgagctcGGCCTCCTGACCTCCTCGTCTT	
ARFTF17-Flag-F	ggaggtcaggaggccgagctcATGgactacaaggaccatgacggtgactacaaggaccatgacattgactacaaggatgacgatgacaagggaggaggaAAGGAGGCGGGCGAGGAGAG	
ARFTF17-CDS-R	cgatcggggaaattcgagctcTCAAGCCCGCTCGACCAT	
GFP-CDS-F	ggaggtcaggaggccgagctcATGGTGAGCAAGGGCGAGGA	
GFP-CDS-R	cgatcggggaaattcgagctcTTACTTGTACAGCTCGTCCA	
MYB40-FlagF	aggtcgactctagaggatccATGgactacaaggaccatgacggtgactacaaggaccatgacattgactacaaggatgacgatgacaagggaggaggaGGGAGGGCGCCGTGCTGCGAGA	
MYB40CDS-R	ggggaaattcgagctcCTAGAGATTGTCCAGGAAGAAG	
ARFTF17Pro-F1	ATCACCACAACAAGGTAGAGGC	Identification of transgenic plants
ARFTF17-R	AAATGTGGCGGAACTTCCAGAC	
GFP-R1	AAGTTCACCTTGATGCCGTTCTT	
ubi1-F	TTAGCCCTGCCTTCATACGC	
MYB40-R	CAGGTGCGAGTTCCAGTAGTTTT	
RNAi-R	AATTGACGCAAGGAGAAACGT	
ARFTF17-GFPF	gactgagctcggtacccggggatccATGAAGGAGGCGGGCGAGGAGAG	Subcellular localization
ARFTF17-GFPR	ctcgcccttgctcaccatgtcgacAGCCCGCTCGACCATGTATT	
ARFTF17-PET30F	acaaggccatggctgatatcggatccAAGGAGGCGGGCGAGGAGAG	Recombinant protein preparation
ARFTF17-PET30R	gctcgagtgcggccgcaagcttgtcgacAGCCCGCTCGACCATGTATTC	
MYB40-pCOLDF	cgcggatccATGGGGAGGGCGCCGTGCTGCGAGA	
MYB40-pCOLDR	ccggaattcCTAGAGATTGTCCAGGAAGAAG	
ARFTF17-62SKF	gctctagaactagtggatccATGAAGGAGGCGGGCGAGGAG	Dual-luciferase reporter assay
ARFTF17-62SKR	tcctgcagcccgggggatccTCAAGCCCGCTCGACCATGTAT	
MYB40-62SKF	gctctagaactagtggatccCTCGCCCTTTTCACTTTTCAG	
MYB40-62SKR	tcctgcagcccgggggatccACCTACAAGTACCGGATCA	

	AATTAA	
CHS-LUCF	cggtatcgataagcttAGCTTAATGGATGCCGACAGT	
CHS-LUCR	aattcgatctccaccgcggAGGAACCAAGCAAGCAAGCAC	
DFR-LUCF	cggtatcgataagcttAAAGTGTTGTACTACTTTTGCA	
DFR-LUCR	aattcgatctccaccgcggCAGACACTTGCTTTGCTTCTTTAG	
PIN1-LUCF	cggtatcgataagcttTTTCAGATTTGCCTTATTCAC	
PIN1-LUCR	aattcgatctccaccgcggCACCAAGAAACACTTGCGAGC	
ARFTF17-771F	cacgggggacgagctcggtaccATGAAGGAGGCGGGCGAGGAGAG	Luciferase complementation imaging assay
ARFTF17-771R	acgcgtacgagatctggtcgacAGCCCGCTCGACCATGTAT	
MYB40-772F	cgtacgcgtcccggggcggtaccATGGGGAGGGCGCCGTGCTGC	
MYB40-772R	acgaaagctctgcaggtcgacCTAGAGATTGTCCAGGAAGAAGA	
MYB40-YC-F	CGACTCTAGGAGCTCGGTACCCGGGATGGGGAGGGCGCCGTGCTGCGAGA	Bimolecular fluorescence complementation assays
MYB40-YC-R	GAACATCGTATGGGTACATACTAGTGAGATTGTCCAGGAAGAAG	
ARFTF17-YN-F	GATTTCTGAGGAGGATCTTCCCGGGAAGGAGGCGGGCGAGGAGAG	
ARFTF17-YN-R	AAGCAGGGCATGCCTGCAGGTCGACTCAAGCCCGCTCGACCATGTAT	
CHS-EMSA-F3	ACCCAACTAACCCCGGCGC	EMSA
CHS-EMSA-R3	ACCGGGTTAGGTCAGTTGG	
DFR-EMSA-F2	GCAGAGATATGCCGGTAGG	
DFR-EMSA-R2	GCTGCTGCTCCAGTTCCGA	
PIN1-EMSA-F	AATGCACAATCAAATAAATACTCAA	
PIN1-EMSA-R	ATTTCTTCAAAACTGAAAATTGTTG	
DR5-EMASF	TTCACCTCGACGGTATCGCG	
DR5-EMASR	GAGGCCTGCCCCCTTTTGTCTC	
P3(2X)-EMSAF	AAGGGAGACAACTTGTCTCCCAAAGGGAGACAACTTGTCTCCCAAAGGGAGACAACTTGTCTCCCA	
P3(2X)-EMSAR	TGGGAGACAAGTTGTCTCCCTTTGGGAGACAAGTTGTCTCCCTTTGGGAGACAAGTTGTCTCCCTT	
