# Supplementary material for: An ARF gene mutation creates flint kernel architecture in dent maize
Source: Nat Commun. 2024 Mar 22;15:2565. doi: 10.1038/s41467-024-46955-9 (PMC10960022; doi:10.1038/s41467-024-46955-9)
Supplement: Supplementary file 3 — Description of Additional Supplementary Files [file 41467_2024_46955_MOESM3_ESM.pdf]

<u>Description of Additional Supplementary Files</u>

Supplementary Data 1: DEGs of B73 vs *fka1-1* in pericarp at 12, 20 and 30 DAP.

Supplementary Data 2: DEG intersection of B73 vs *fka1-1* in pericarp at 12, 20 and 30 DAP.

Supplementary Data 3: FPKM values of DEGs in phenylpropanoid pathway in B73 and *fka1-1* pericarps at 12, 20 and 30 DAP.

Supplementary Data 4: RT-qPCR data of DEGs in phenylpropanoid pathway in B73 and *fka1-1* pericarps at 15 and 25 DAP.

Supplementary Data 5: Differential metabolites in pericarp of B73 vs *fka1-1* at 15 and 25 DAP.

Supplementary Data 6: Interaction of differential metabolites in pericarp of B73 vs *fka1-1* at 15 and 25 DAP.
