# Supplementary material for: An ARF gene mutation creates flint kernel architecture in dent maize
Source: Nat Commun. 2024 Mar 22;15:2565. doi: 10.1038/s41467-024-46955-9 (PMC10960022; doi:10.1038/s41467-024-46955-9)
Supplement: Supplementary file 10 — Reporting Summary [file 41467_2024_46955_MOESM10_ESM.pdf]

nature portfolio

Corresponding author(s): Yongrui Wu

Last updated by author(s): Feb 3, 2024

Reporting Summary

Nature Portfolio wishes to improve the reproducibility of the work that we publish. This form provides structure for consistency and transparency in reporting. For further information on Nature Portfolio policies, see our Editorial Policies and the Editorial Policy Checklist.

Statistics

For all statistical analyses, confirm that the following items are present in the figure legend, table legend, main text, or Methods section.

n/a	Confirmed	
☐	☒	The exact sample size (*n*) for each experimental group/condition, given as a discrete number and unit of measurement
☐	☒	A statement on whether measurements were taken from distinct samples or whether the same sample was measured repeatedly
☐	☒	The statistical test(s) used AND whether they are one- or two-sided<br>*Only common tests should be described solely by name; describe more complex techniques in the Methods section.*
☒	☐	A description of all covariates tested
☒	☐	A description of any assumptions or corrections, such as tests of normality and adjustment for multiple comparisons
☐	☒	A full description of the statistical parameters including central tendency (e.g. means) or other basic estimates (e.g. regression coefficient) AND variation (e.g. standard deviation) or associated estimates of uncertainty (e.g. confidence intervals)
☐	☒	For null hypothesis testing, the test statistic (e.g. *F*, *t*, *r*) with confidence intervals, effect sizes, degrees of freedom and *P* value noted<br>*Give P values as exact values whenever suitable.*
☒	☐	For Bayesian analysis, information on the choice of priors and Markov chain Monte Carlo settings
☒	☐	For hierarchical and complex designs, identification of the appropriate level for tests and full reporting of outcomes
☒	☐	Estimates of effect sizes (e.g. Cohen's *d*, Pearson's *r*), indicating how they were calculated

Our web collection on statistics for biologists contains articles on many of the points above.

Software and code

Policy information about availability of computer code

Data collection	Illumina novaseq6000 (BSA sequencing); Illumina novaseq6000 (RNA-seq); Bio-Rad CFX Manager v3.1 (RT-qPCR data); Tanon-5200Tanon Science and Technology (immunoblot image; LCI); luminometer (Promega 20/20) (DLR); Starion FLA-9000 instrument (FujiFilm, Japan) (EMSA); LSM880 Zeiss, Jena, Germany(confocal microscope); UPLC (Ultra Performance Liquid Chromatography UPLC)-MS/MS (Tandem mass spectrometry) using Analyst 1.6.3 software (AB Sciex) (Metabolome analysis); UPLC (Measurement of auxin content).
Data analysis	GraphPad Prism v8 and Microsoft Excel 2016 (two-side Student's t-test, one-way ANOVA and Tukey's test); Tools in (www.omicshare.com/tools/) for RNA-seq data and metabolome analysis.

For manuscripts utilizing custom algorithms or software that are central to the research but not yet described in published literature, software must be made available to editors and reviewers. We strongly encourage code deposition in a community repository (e.g. GitHub). See the Nature Portfolio guidelines for submitting code & software for further information.

Data

Policy information about availability of data

All manuscripts must include a data availability statement. This statement should provide the following information, where applicable:

- Accession codes, unique identifiers, or web links for publicly available datasets
- A description of any restrictions on data availability
- For clinical datasets or third party data, please ensure that the statement adheres to our policy

All data are available in the main text or the supplementary materials. Data for each figure in this paper are provided in the Supplementary Data files and a Source Data file. Source data are provided with this paper.

Research involving human participants, their data, or biological material

Policy information about studies with human participants or human data. See also policy information about sex, gender (identity/presentation), and sexual orientation and race, ethnicity and racism.

Reporting on sex and gender	n/a
Reporting on race, ethnicity, or other socially relevant groupings	n/a
Population characteristics	n/a
Recruitment	n/a
Ethics oversight	n/a

Note that full information on the approval of the study protocol must also be provided in the manuscript.

Field-specific reporting

Please select the one below that is the best fit for your research. If you are not sure, read the appropriate sections before making your selection.

☒ Life sciences ☐ Behavioural & social sciences ☐ Ecological, evolutionary & environmental sciences

For a reference copy of the document with all sections, see nature.com/documents/nr-reporting-summary-flat.pdf

Life sciences study design

All studies must disclose on these points even when the disclosure is negative.

Sample size	No statistical methods were used to predetermine sample size. Sample sizes were estimated based on preliminary experiments and previously published results (Huang et al., 2022, Nature). We made the effort to achieve a large sample size in field trial which proved to be sufficient to reproducibly observe statistically significant differences, sample size is stated in each panel. For screening IKA1 from an EMS-induced B73 mutant, we yielded more than 2000 M1 ears. Ten M2 seeds from each M1 ear were planted and the plant that grew were self-pollinated, producing only about 15,000 M2 ears.To clone the IKA1 gene, we created an F2 population from the cross between B73 and IKA1, and obtained dent and flint like ears of 392 and 110. Sample size of all experiments were showed in the figure legends and source data.
Data exclusions	No data was excluded from the analyses.
Replication	All experiments in this study were repeated independently at least three times. This information is shown in figure legends.
Randomization	All samples were arranged randomly into experimental groups.
Blinding	For molecular biology experiments, bias could not be introduced since samples were treated identically and collected randomly. No analyses required being blind to groups.

Reporting for specific materials, systems and methods

We require information from authors about some types of materials, experimental systems and methods used in many studies. Here, indicate whether each material, system or method listed is relevant to your study. If you are not sure if a list item applies to your research, read the appropriate section before selecting a response.

Materials & experimental systems

n/a	Involved in the study
☐	☒ Antibodies
☒	☐ Eukaryotic cell lines
☒	☐ Palaeontology and archaeology
☒	☐ Animals and other organisms
☒	☐ Clinical data
☒	☐ Dual use research of concern
☐	☒ Plants

Methods

n/a	Involved in the study
☒	☐ ChIP-seq
☐	☒ Flow cytometry
☒	☐ MRI-based neuroimaging

Antibodies

Antibodies used

Anti-ARFTF17 (made by ABclonal, 1:1000); Anti-FLAG (Sigma, A8592, 1:1000); Anti-ACTIN (Abmart, catalog number M20009L, 1:1000); anti-rabbit-HRP (Abmart, catalog number: M21002L; 1:5000); anti-mouse IgG-HRP (Abmart, catalog number M21001L; 1:5000) for immunoblot analysis.

Validation

ARFTF17 protein fragment from the 480th to 644th amino acid was used to make antibodies by ABclonal (Wuhan, China). B73 and IKA1 were used to validate anti-ARFTF17 antibodies. Mouse monoclonal ACTIN antibody (Abmart, catalog number M20009L), a secondary antibody, repeatedly used in our previous research (Huang et al., Nature, 2022). The monoclonal antibody anti-FLAG (Sigma, A8592) binds to fusion proteins containing a FLAG peptide sequence at the N-terminus of ARFTF17. The specific band was shown in the WB using pericarp protein of ARFTF17pro:Flag-ARFTF17 in Extended Data c, but without in WT B73 .

Dual use research of concern

Policy information about dual use research of concern

Hazards

Could the accidental, deliberate or reckless misuse of agents or technologies generated in the work, or the application of information presented in the manuscript, pose a threat to:

No	Yes
☒	☐ Public health
☒	☐ National security
☒	☐ Crops and/or livestock
☒	☐ Ecosystems
☒	☐ Any other significant area

Experiments of concern

Does the work involve any of these experiments of concern:

No	Yes
☒	☐ Demonstrate how to render a vaccine ineffective
☒	☐ Confer resistance to therapeutically useful antibiotics or antiviral agents
☒	☐ Enhance the virulence of a pathogen or render a nonpathogen virulent
☒	☐ Increase transmissibility of a pathogen
☒	☐ Alter the host range of a pathogen
☒	☐ Enable evasion of diagnostic/detection modalities
☒	☐ Enable the weaponization of a biological agent or toxin
☒	☐ Any other potentially harmful combination of experiments and agents

Plants

Seed stocks	EMS mutant library MEMD (http://elabcaas.cn/memd/public/index.html#/) under the accession number EMS4-0718ab and EMS4-0b3dd4 respectively.
Novel plant genotypes	The ARFTF mutants were created in our lab using CRISPR/Cas9 in the High-II hybrid (B x A) via Agrobacterium-mediated transformation. The specific single, double, triple and quadruple ARFTF mutations were introgressed into B73 for four generations by backcrossing. Constructs ARFTF17Pro:GFP, ARFTF17Pro:ARFTF17 andMYB40-OE were transformed into B73 via Agrobacterium-mediated transformation by Wimi Biotechnology (Jiangsu).
Authentication	fka1-1, fka1-2 and pin1 mutations were identified by PCR amplification using specific primers. ARFTF CRISPR mutations were identified using the specific primers for each ARFTF genes; then the sequences were analyzed through the website (http://skl.scau.edu.cn/dsdecode/). The transgenic plants were confirmed by PCR amplification. All primers used are listed in Supplementary Table 1.

Flow Cytometry

Plots

Confirm that:

☐ The axis labels state the marker and fluorochrome used (e.g. CD4-FITC).

☐ The axis scales are clearly visible. Include numbers along axes only for bottom left plot of group (a 'group' is an analysis of identical markers).

☐ All plots are contour plots with outliers or pseudocolor plots.

☒ A numerical value for number of cells or percentage (with statistics) is provided.

Methodology

Sample preparation	Pericarps were put in a 5-cm plastic petri dish, and added 1 ml of ice-cold Galbraith's extraction buffer (45 mM MgCl2, 20 mM MOPS, 30 mM sodium citrate, 0.1% [v/v] Triton X-100, 0.05 mM sodium metabisulfite, 0.5% [v/v] -Mercaptoethanol, pH 7.0)50, followed by rapid chopping with a new razor blade.
Instrument	CytoFLEX LX (Beckman Coulter)
Software	Data were collected and analyzed using CytExpert (Beckman Coulter) software.
Cell population abundance	The sample was filtered through a 40-mm nylon mesh into a 1.5-ml sample tube. Averagely, about 115000 events were detected in a sample, and 25000 cells were detected.
Gating strategy	The sample was added to DAPI buffer (5 µg/ml) to mark the cell nucleus. We used DAPI to identify cells by the accumulation of signals in PB450/SSC to to gate the flow cytometry.

☒ Tick this box to confirm that a figure exemplifying the gating strategy is provided in the Supplementary Information.
